# Pathogenic landscape shaped by cerebral amyloid oligomers

**DOI:** 10.4103/NRR.NRR-D-25-00557

**Published:** 2025-06-19

**Authors:** Yujing Huang, Mengze Xu, Zhen Yuan, Pu Chun Ke

**Affiliations:** Faculty of Health Sciences, University of Macau, Macao Special Administrative Region, China; Department of Biology, Faculty of Arts and Sciences, Beijing Normal University, Zhuhai, Guangdong Province, China; Center for Cognitive and Brain Sciences, University of Macau, Macao Special Administrative Region, China; Drug Delivery, Disposition and Dynamics, Monash Institute of Pharmaceutical Sciences, Monash University, Parkville, VIC, Australia

**Amyloid oligomers, a brief history:** Amyloid diseases encompass a range of human neurological, systemic, and metabolic disorders, characterized by the common feature of amyloid fibril and plaque deposition, either intracellularly or extracellularly. Among them, Alzheimer’s disease (AD)—manifested by memory loss and cognitive decline—and Parkinson’s disease (PD)—underlined by impaired dopamine release and motor dysfunction—are the two most prevalent forms of neurodegenerative conditions that have rapidly become global epidemics. Type 2 diabetes, conversely, is a prevalent metabolic disorder underpinned by pancreatic beta cell loss, elicited primarily by the aggregation and toxicity of human islet amyloid peptide.

In 1992, Hardy and Higgins put forth the amyloid cascade hypothesis, which depicts the self-assembly of amyloid–beta (Aβ) peptide into fibrils through the dynamic process of amyloidogenesis. Apparently, this paradigm drew its inspiration from the principles of polymerization in polymer science and molecular cell biology, where the sequential addition of monomers gives rise to linear elongation or fibrillization driven by thermodynamics and kinetic factors, with the end products of amyloid fibrils designated as the primary toxic culprits.

In more recent years, amyloid oligomers — small globular, annular and curvilinear (the latter also referred to as protofibrils) intermediates rendered by early-stage protein aggregation — have been implicated as the most toxic species, superseding mature amyloid fibrils in pathological relevance. This shift in perspective is supported by a growing body of compelling evidence. In 2008, Shankar et al. isolated Aβ dimers directly from the brains of AD subjects through centrifugation and immunoprecipitation, linking them to synaptic plasticity deficits and memory loss in mice. Using a primary neuronal culture model, Sandberg et al. (2022) demonstrated that an oligomer-specific antibody (ALZ-201) effectively neutralized the toxic effects of AD brain extracts. Additionally, an α-sheet capture agent successfully detected Aβ oligomers in the plasma of patients with mild cognitive impairment and AD (Shea et al., 2022). Physicochemical and neuropathological analyses have also implicated soluble alpha-synuclein (αS) oligomers as highly toxic, with their accumulation in the hippocampus and association with cortical regions in PD brains spelling a central role for oligomers in neurodegeneration (Emin et al., 2022).

**Biophysical origins of amyloid oligomer toxicity:** While monomeric amyloid proteins such as Aβ, αS, and human islet amyloid peptide are known to perform certain physiological functions and amyloid fibrils have been proposed to possess a controversial toxic-protective duality, amyloid oligomers are predominantly viewed as pathogenic — or, at best, nonfunctional. Biophysically, these soluble oligomers expose hydrophobic surface pockets due to irregular monomer packing, rendering them prone to aberrant interactions with amphiphilic cell membranes, proteins, and small molecules in their local environment. Such interactions can promote further monomer-to-oligomer conversion and induce membrane poration, leading to metal ion influxes that stress the endoplasmic reticulum and activate the unfolded protein response. If sustained, this stress can escalate into the production of reactive oxygen species (ROS), mitochondrial damage, and cellular inflammation. Moreover, oligomers can impair the immune response of the central nervous system by diminishing the capacity of microglia to clear amyloid aggregates, thereby contributing to proteasome dysfunction, apoptosis, and progressive neurodegeneration (Zott et al., 2019; Tang et al., 2025).

From a free-energy perspective, amyloid oligomers reside in quasi-stable energy minima (Tang et al., 2025), making them highly reactive and prone to interacting with local biomolecules and organelles in an effort to lower their surface energy. This intrinsic instability drives their structural reorganization, allowing them to dynamically reshape cellular homeostasis and protein synthesis — often with pathogenic consequences. In contrast, amyloid fibrils represent a thermodynamic end state, occupying the global free-energy minimum due to the tight packing of monomers into a cross-β sheet backbone. The lower surface hydrophobicity per monomer of amyloid fibrils renders them less reactive than oligomers. Over time, amyloid fibrils can evolve into plaques through hydrogen bonding, hydrophobic interactions, and π–π stacking. Owing to their waxy, hydrophobic surfaces, amyloid fibrils can extract lipids from cellular membranes, thereby increasing membrane fluidity. Unlike soluble oligomers, insoluble amyloid fibrils can spontaneously acquire a protein “corona” from their surroundings, gaining a new biological identity while reducing their intrinsic toxicity (Tang et al., 2025).

The concept of secondary nucleation in amyloidogenesis refers to the process in which monomeric proteins are converted into amyloid fibrils through catalysis at the hydrophobic surfaces of pre-existing fragmented fibrils or seeds. This mechanism is significantly faster and more efficient than the stepwise monomer addition seen in primary nucleation and presents a promising avenue for therapeutic interventions aimed at suppressing the formation of toxic oligomers. Beyond its role in fibrillization, secondary nucleation also carries important implications for understanding amyloid remodeling and clearance. Notably, secondary nucleation entails new insights into the phenomenon of cross-seeding — where amyloid proteins of different origins influence each other’s aggregation — which may underlie pathological interactions between diseases such as AD, PD, and type 2 diabetes.

In addition to primary and secondary nucleation of the revamped amyloid hypothesis, recent theoretical advances have highlighted the role of liquid–liquid phase separation (LLPS) in amyloidogenesis. LLPS is a biophysical process well established in cell biology, whereby proteins and nucleic acids de-mix from their surroundings to form dynamic, membrane-less condensates that facilitate both physiological functions and pathological outcomes. In the context of amyloid proteins, LLPS leads to elevated local concentrations and emergence of new dynamic interfaces, thereby promoting nucleation and aggregation. This framework has, for instance, shed light on the amyloidogenesis of TAR DNA-binding protein 43 (TDP-43), whose low-complexity domain drives LLPS and pathological aggregation in sporadic amyotrophic lateral sclerosis (Tang et al., 2025).

**Vascular damage induced by amyloid oligomers:** Amyloid deposition in AD is strongly correlated (80%–90%) with cerebral amyloid angiopathy, suggesting a potential causative link between amyloid pathology and vascular dysfunction. Indeed, disruption of the blood-brain barrier and damage to cerebral vasculature are hallmark features arising from amyloid accumulation, although their precise working mechanisms remain incompletely understood. ROS, generated by amyloidogenesis, is implicated in endothelial inflammation and alterations to the cytoskeletal protein network. These changes often result in the disruption of tight and adherens junctions to elevate endothelial permeability. For example, exposure of blood vessels to 10 μM Aβ for 15 minutes induced excessive superoxide radical production, leading to lipid peroxidation and structural damage to the endothelium (Thomas et al., 1996). Furthermore, in both human and mouse models of AD, cerebral capillaries exposed to Aβ oligomers (up to 80 nM for 60 minutes) exhibited constriction at pericyte sites via ROS-mediated mechanisms, resulting in a roughly 50% reduction in cerebral blood flow — an effect that promoted hypoxia and neurodegeneration (Nortley et al., 2019). Despite these findings, the connections between amyloidogenesis in neurological disorders and vascular health remain complex and scientifically arresting.

Recently, we have discovered that the oligomers and seeds of anionic Aβ and αS, but not the cationic human islet amyloid peptide, can permeate the paracellular space of endothelial monolayers, forming microscopic gaps both *in vitro* and *in vivo* (Li et al., 2024). This phenomenon, termed amyloid protein-induced endothelial leakiness (APEL), arises from direct molecular interactions between negatively charged amyloid aggregates (< 100 nm in size) and apposing vascular endothelial-cadherin dimers that maintain the integrity of the cerebral vasculature. Unlike previously described mechanisms involving oxidative stress, inflammation, or apoptosis, APEL represents a distinct biophysical process, accompanied by aberrant adherens junction signaling but independent of endocytosis, ROS production, or cell death (Li et al., 2024).

**Figure 1:**
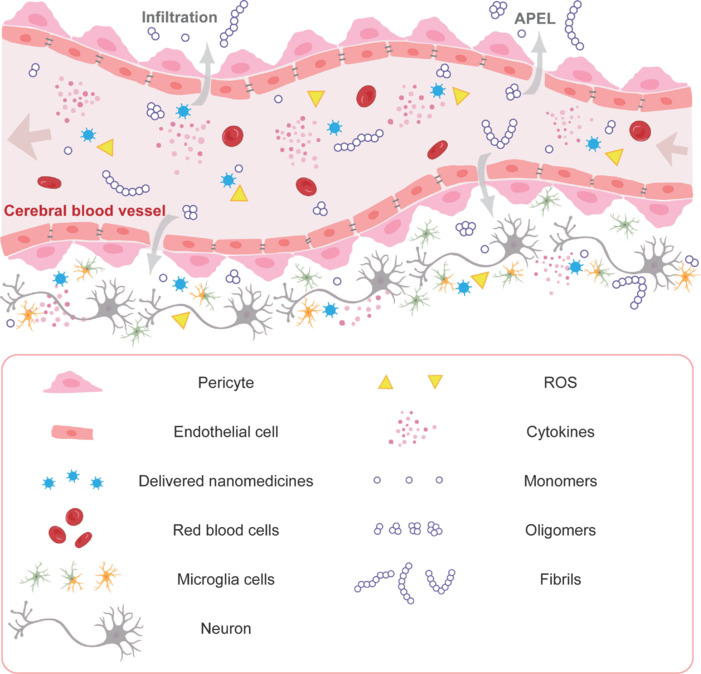
Schematic illustration of cerebral vascular damage and neurodegeneration elicited by amyloidogenesis and their potential mitigation by delivered nanomedicines. APEL: Amyloid protein-induced endothelial leakiness; ROS: reactive oxygen species.

The identification of APEL carries broad biological and pathological implications. It offers a new mechanism for the systemic dissemination and cross-talks of amyloid pathologies in AD, PD, and type 2 diabetes (**Figure 1**). Moreover, APEL may foster vascular infiltration observed in systemic amyloidosis, where the conformation of the causative immunoglobulin light chain aggregates remains vague in literature. By disrupting neuronal adhesion molecular complexes, APEL may further compromise synaptic neurotransmission, exacerbating the pathophysiology of AD and PD.

**Mitigation strategies against amyloidosis and oligomer-mediated cerebral vascular damage:** Mitigating the adverse effects of amyloid oligomers can, in principle, be realized by outcompeting peptide-peptide interactions in amyloidogenesis with targeted peptide-antagonist interactions. Since 1997, a range of strategies have been devised, involving the antagonists of small molecules (e.g., curcumin, epigallocatechin gallate), peptides (e.g., the beta-sheet “breaker” KLVFF, inspired by the amyloidogenic core of Aβ), metal ions (via chelation with amyloid fibrils), monoclonal antibodies (e.g., lecanemab, which targets protofibrils, a curvilinear toxic form of oligomers), or nanoparticles/composites (e.g., gold nanoparticles stabilized by milk protein β-casein). While these strategies have shown a remarkable capability in mitigating amyloidogenesis, their *in vivo* execution is hindered by poor delivery, post-translational modifications of amyloid proteins, as well as the formation of a heterogeneous and dynamic protein corona acquired over time by amyloid protein aggregates.

Future strategies for mitigating cerebral amyloid oligomers and their induced vascular damage should address the following challenges: (a) enhancing blood–brain barrier translocation *in vivo*; (b) improving the targeting and clearance of amyloid oligomers, protofibrils, fibrils, and their fragments/seeds within the central nervous system, including the cerebral vasculature; (c) sequestering ROS and inflammatory cytokines generated during amyloidogenesis and cerebral vascular damage (infiltration and leakiness) (**Figure 1**); (d) stabilizing vascular endothelial-cadherin interactions and mitigating APEL by designing oligomer-specific neutralizing agents; (e) restoring the phagocytic capacity of microglia and astrocytes; and (f) reestablishing cerebral blood flow and cellular homeostasis to support neuronal regeneration. To this end, biocompatible and biodegradable multimodal nanomedicines or cocktails of single-purpose nanomedicines to leverage the following properties: (a) efficient translocation across biological barriers, including the blood–brain barrier; (b) nonspecific hydrophobic interactions and H-bonding with amyloid oligomers and protofibrils to suppress amyloidogenesis; (c) specific targeting of cerebral amyloid proteins, particularly amyloid oligomers; (d) remodeling and clearance of amyloid fibrils and plaques; (e) surface functionality-mediated targeting and repair of neuronal, immune and endothelial cells within the central nervous system; and (f) preservation of the gut-brain axis by mitigating the impact of endogenous and exogenous toxins and ROS may offer new therapeutic outcomes against neurodegeneration and neurological disorders.

As a parting note, while most mechanistic insights discussed in this perspective, and in the broader literature, have been derived from Aβ and αS models, it is important to recognize that amyloid aggregation, cerebrovascular damage, and immune activation in AD and vascular dementia often involve other proteins, such as tau and medin. These interconnected processes deserve thorough future investigation to fully elucidate the pathogenic landscape shaped by cerebral amyloid oligomers and to inform more effective strategies for intervention in neurological disorders.

*We acknowledge Professors Colin Masters and Scott Ayton from Florey Institute, The University of Melbourne, Australia for discussions.*

*This work was supported by the National Key Research and Development Program of China (2021YFA1200900 and 2022YFC2409700) and the Distinguished Visiting Scholar Program with the University of Macau (to PCK).*
